# Recurrent Neck Mass: A Case Report

**DOI:** 10.7759/cureus.22098

**Published:** 2022-02-10

**Authors:** Dylan Z Erwin, David Lesko, Jay K Ferrell

**Affiliations:** 1 Otolaryngology - Head and Neck Surgery, University of Texas Health Science Center at San Antonio, San Antonio, USA

**Keywords:** branchial arch malformation, recurrent neck mass, congenital neck mass, embryology, branchial anomaly

## Abstract

Fourth branchial cleft anomalies are an exceptionally rare cause of recurrent neck mass in pediatric and adult patients. In this report, we present a case of an infected fourth branchial cleft cyst in a 20-year-old woman that presented with recurrent throat pain and deep neck abscesses. After undergoing repeated incision and drainage procedures, the patient underwent definitive management with direct laryngoscopy, ablation of the left pyriform sinus tract, left hemithyroidectomy, and excision of the branchial anomaly without evidence of recurrence. In addition to diagnosis and management, this case report highlights the unique anatomical relationship between fourth branchial anomalies and the pyriform fossa as well as the superior and recurrent laryngeal nerves.

## Introduction

Branchial anomalies are believed to arise from incomplete obliteration of the branchial elements. They may manifest as cysts, fistulas, or sinus tracts. While second branchial anomalies are the most common (representing over 95%), fourth branchial anomalies are exceedingly rare (less than 1%) [1,2].

As there are no pathognomonic clinical or radiographic features, diagnosing branchial cleft anomalies can be challenging and is based on patient history and examination [3]. When compared to second branchial anomalies, fourth branchial anomalies are located lower in the anterior neck. In addition, unlike third branchial anomalies, they terminate at the apex of the piriform sinus and are more intimately associated with the recurrent laryngeal nerve. Given their rare presentation, they are often misdiagnosed as thyroiditis or visceral space abscesses [2].

We report the case of an infected fourth branchial cleft cyst, which presented as a recurrent deep neck infection in a young adult female treated at a large academic center. This case demonstrates the diagnostic challenge of these anomalies and the keys to their successful management. A focused literature review highlighting the history surrounding these lesions is also provided. 

## Case presentation

A 20-year-old female presented in early 2018 with a two-year history of recurrent left neck abscesses and treatment with antibiotics and steroids. She had recently undergone an incision and drainage procedure of a left deep neck abscess at another facility, but presented to our emergency department with recurrence several weeks later. Due to concerns for a possible congenital anomaly, she underwent percutaneous drainage by interventional radiology with resolution of symptoms. She remained asymptomatic with a normal physical exam through September 2018.

**Figure 1:**
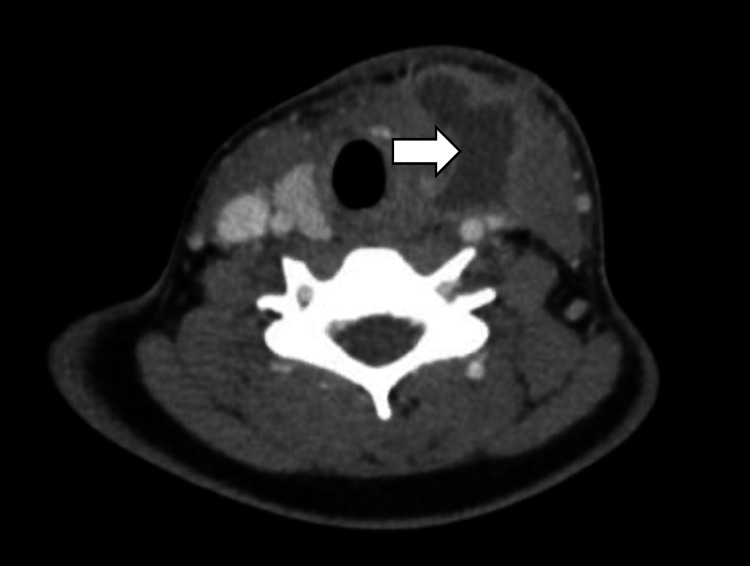
CT neck with contrast White arrow demonstrates the left fourth branchial cleft cyst tracking towards the left pyriform sinus.

She then re-presented to us in October 2018 with throat pain and left lateral neck swelling and induration. CT neck with contrast demonstrated an ill-defined heterogeneous lesion of the left paratracheal region with a fistulous tract extending toward the left pyriform sinus (Figure 1). These findings, along with her history and physical examination, were suspicious for infected left fourth branchial cleft cyst.

The patient was admitted for intravenous (IV) antibiotics and repeat drainage. She was subsequently taken to the operating room (OR) for incision and drainage and direct laryngoscopy. Intraoperatively, an opening to the tract in the left pyriform sinus was identified. However, it was not cauterized at that time due to significant edema. Intraoperative cultures were obtained, and the patient was subsequently given culture-directed antibiotics.

**Figure 2:**
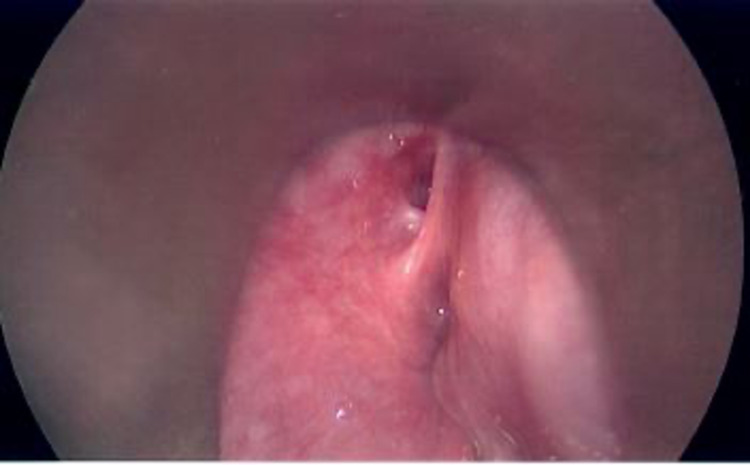
Direct laryngoscopy showing opening at the apex of the left pyriform sinus.

After improvement in her acute inflammatory symptoms, she was taken back to the OR for definitive excision of the branchial cleft cyst and endoscopic management of the fistulous tract. Direct laryngoscopy revealed a well-defined sinus tract opening in the apex of the left pyriform sinus (Figure 2) that was cauterized endoscopically via coblation.

**Figure 3:**
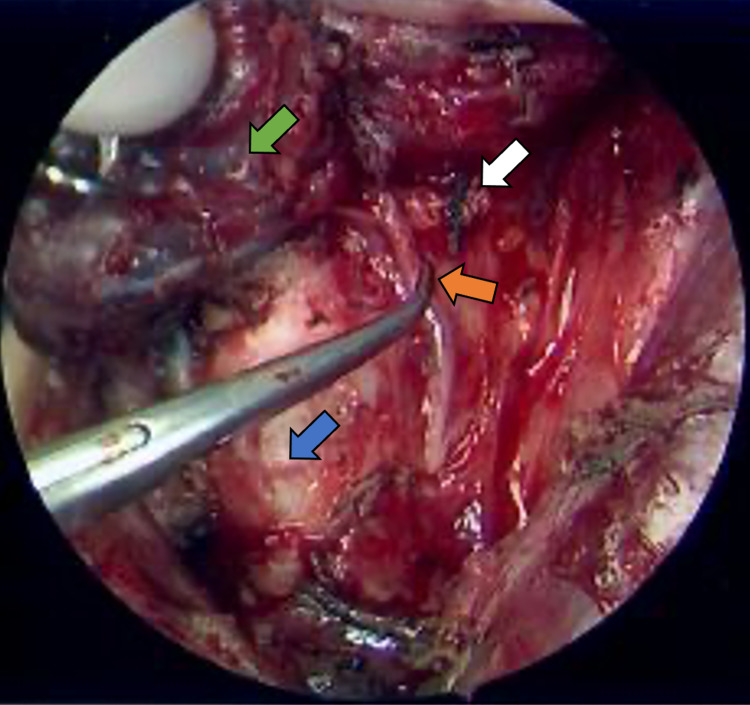
Intraoperative image Intraoperative view of the fourth branchial cleft cyst (white arrow) with fistulous tract coursing beneath the recurrent laryngeal nerve (orange arrow) toward the thyroid cartilage/pyriform sinus apex. Green arrow and blue arrow denote thyroid and trachea, respectively.

Subsequent open neck dissection was difficult due to scarring and obliteration of natural tissue planes as a result of her recurrent infections. At the level of the cricoid cartilage, medial to the internal carotid artery and jugular vein, an ill-defined soft tissue density, suspicious for the remnant branchial cleft cyst, was identified. This specimen was tightly adherent to adjacent strap musculature which necessitated resection with partial muscle sacrifice. As the superior thyroid pole was transected, we identified a distinct fistulous tract connecting the mass to the undersurface of the thyroid cartilage in the direction of the left pyriform sinus. The tract traveled in tandem with the recurrent laryngeal nerve (Figure 3). This tract was dissected along its full course to the undersurface of the thyroid cartilage, suture ligated, and the extra-laryngeal specimen was resected.

Surgical pathology demonstrated suspected branchial cleft anomaly and benign thyroid tissue with adherent soft tissue, consistent with a tract. At most recent follow up nine months post-operatively, the patient has healed well and not experienced any pain or infection.

## Discussion

Branchial anomalies are an important consideration in the differential of recurrent pediatric and adult neck masses. As demonstrated with this case, fourth branchial cleft cysts are commonly left-sided and course from the apex of the pyriform fossa to the upper aspect of the left thyroid lobe. As a result, patients can present with a recurrent abscess in the low anterior neck or recurrent suppurative thyroiditis [2].

In the past, it has been debated as to whether third and fourth branchial anomalies represent two distinct entities. The terms "pyriform sinus tract” or “pyriform sinus lesion” have been applied to both. However, it has more recently been accepted that they indeed are different. Third branchial anomalies originate from the base of the pyriform sinus and pass above the superior laryngeal nerve to enter the larynx via the thyrohyoid membrane. In contrast, fourth branchial anomalies originate from the apex of the pyriform sinus and run in parallel with the recurrent laryngeal nerve, as demonstrated in this case [4]. 

The tandard curative treatment for fourth branchial cleft anomalies is surgical excision. However, due to the anatomical course, complete excision is often difficult [1]. In this case, excision was complicated by obliterated tissue planes from recurrent infection and tract proximity to the recurrent laryngeal nerve. Hemithyroidectomy was performed to ensure complete resection and decrease the risk of recurrence, as well as safe identification and preservation of the recurrent laryngeal nerve. In addition, endoscopic cauterization of the pyriform sinus opening was employed to prevent a recurrence. While no complications were observed, vocal cord paralysis, salivary fistula, and recurrence have been reported, particularly in children eight or younger [5,6]. Some authors advocate delaying open neck surgery in young children and performing only endoscopic cauterization initially to decrease complication rates and simplify post-operative care [5]. Complete removal of the anomalous tract is key to preventing disease recurrence, and success can be measured by close follow-up after operative repair [7,8]. 

## Conclusions

This case illustrates the presentation, diagnosis, and management of a rare branchial anomaly presenting as recurrent neck infections in an adult. Fourth branchial anomalies are often misdiagnosed due to their rarity and lack of clinical or imaging features. Accurate diagnosis relies on keen attention to the history, thorough knowledge of the anatomy, and a high index of suspicion. This case highlights the unique anatomical relationship fourth branchial anomalies have with the pyriform sinus and the superior and recurrent laryngeal nerves. Successful, definitive management requires complete excision of left branchial cleft cyst and ablation of the associated pyriform sinus tract.
